# Dealing with heterogeneity of treatment effects: is the literature up to the challenge?

**DOI:** 10.1186/1745-6215-10-43

**Published:** 2009-06-19

**Authors:** Nicole B Gabler, Naihua Duan, Diana Liao, Joann G Elmore, Theodore G Ganiats, Richard L Kravitz

**Affiliations:** 1 Center for Healthcare Policy and Research, University of California, Davis, California, USA; 2 Columbia University, New York State Psychiatric Institute, New York, USA; 3 University of California, Los Angeles Neuropsychiatric Institute, California, USA; 4 University of Washington School of Medicine, Seattle, Washington, USA; 5 Department of Medicine, Harborview Medical Center, Seattle, Washington, USA; 6 Department of Family and Preventive Medicine, University of California, San Diego, California, USA; 7 Department of Internal Medicine, University of California, Davis, California, USA

## Abstract

**Background:**

Some patients will experience more or less benefit from treatment than the averages reported from clinical trials; such variation in therapeutic outcome is termed heterogeneity of treatment effects (HTE). Identifying HTE is necessary to individualize treatment. The degree to which heterogeneity is sought and analyzed correctly in the general medical literature is unknown. We undertook this literature sample to track the use of HTE analyses over time, examine the appropriateness of the statistical methods used, and explore the predictors of such analyses.

**Methods:**

Articles were selected through a probability sample of randomized controlled trials (RCTs) published in *Annals of Internal Medicine*, *BMJ*, *JAMA*, *The Lancet*, and *NEJM *during odd numbered months of 1994, 1999, and 2004. RCTs were independently reviewed and coded by two abstractors, with adjudication by a third. Studies were classified as reporting: (1) HTE analysis, utilizing a formal test for heterogeneity or treatment-by-covariate interaction, (2) subgroup analysis only, involving no formal test for heterogeneity or interaction; or (3) neither. Chi-square tests and multiple logistic regression were used to identify variables associated with HTE reporting.

**Results:**

319 studies were included. Ninety-two (29%) reported HTE analysis; another 88 (28%) reported subgroup analysis only, without examining HTE formally. Major covariates examined included individual risk factors associated with prognosis, responsiveness to treatment, or vulnerability to adverse effects of treatment (56%); gender (30%); age (29%); study site or center (29%); and race/ethnicity (7%). Journal of publication and sample size were significant independent predictors of HTE analysis (p < 0.05 and p < 0.001, respectively).

**Conclusion:**

HTE is frequently ignored or incorrectly analyzed. An iterative process of exploratory analysis followed by confirmatory HTE analysis will generate the data needed to facilitate an individualized approach to evidence-based medicine.

## Background

Randomized controlled trials (RCTs) are the cornerstone of evidence-based medicine. Such trials rely on random assignment to alternative treatment groups to control for baseline patient factors that could affect outcomes. The resulting estimate of the average treatment effect is an average of the *individual *treatment effects (ITEs) for participants in the study. While estimates of the *average *treatment effect are generally useful, some treated individuals, both within and outside of clinical trials, will experience more or less benefit than the reported average. Such variation in treatment effect is termed heterogeneity of treatment effects (HTE) [1,2].

HTE may be quantitative (subgroup effects in the same direction as the average effect but varying in magnitude) or qualitative (treatment effects in different directions in different subgroups, where treatment is beneficial in some subgroups and harmful in others). The prevalence of HTE is unknown and perhaps unknowable, but highly variable treatment response rates for many common conditions suggest it is substantial. [3,4] For example, Allen Roses, the vice president of genetics at GlaxoSmithKline, has stated, "Our drugs don't work on most patients" [5]. Several empirical demonstrations of HTE have recently been published, including studies of ischemic stroke [6], risk reduction by carotid endarterectomy [7,8], and diabetes [9]. While qualitative HTE may be uncommon, quantitative HTE should not be dismissed, because even modest variations in the magnitude of net treatment benefits may have important implications for patient care and cost-effectiveness.

HTE can be assessed in several ways. The most direct approach is the n-of-1 clinical trial, which assigns individual patients to receive alternative treatment in a randomly predetermined sequence [10,11]. Results from a series of such trials can be aggregated to assess heterogeneity in the population. However, n-of-1 trials are applicable to a relatively small subset of conditions and treatments [10,11] and are subject to random within-patient variability (thus requiring a careful design and repeated crossovers) [12,13]. A second approach is to stratify patients according to risk of disease-related adverse events [14,15]. A third approach, typically performed for purposes of hypothesis generation rather than testing, entails a careful examination of subgroups within RCTs.

Subgroup analysis can be perilous. Real effects can be missed because of inadequate statistical power [16,17], and reported effects may be spurious because of the performance of multiple statistical tests (13–16) and/or due to random intra-individual variability [12,13]. Random intra-individual variability is especially problematic because it is not possible to estimate this variability in parallel group trials, the most common type of clinical trial design. In parallel group trials, participants are only randomized to one treatment and do not crossover to alternative treatments. As such, it is not possible to estimate any variation that occurs *within *a participant. In recognition of the drawbacks of subgroup analysis, the Consolidated Standards of Reporting Trials (CONSORT) statement warns that subgroup analyses, especially post hoc subgroup comparisons, "do not have great credibility" [18].

On the other hand, it has been claimed that nearly everything we have learned from epidemiology resulted from subgroup analysis [19]. While this conclusion applies most obviously to observational studies, careful scrutiny of subgroup-specific effects in randomized trials has generated important new hypotheses and sometimes directly influenced practice. Nevertheless, subgroup analyses are not always performed correctly. Some studies (e.g. [20-23]) report results *by *subgroups, without any statistical testing or interval estimation for the difference *across *subgroups; these studies do not provide quantitative information on HTE per se. Other studies report p-values corresponding to each subgroup, subsequently claiming that the treatment effect differs across subgroups because it is statistically significant in one subgroup and not in another [24]. However, both treatment effect and sample size influence the p-value, such that similar effect sizes within each subgroup might generate markedly different p-values. Instead of comparing the p-values across subgroups, the appropriate way to identify significant HTE is to make statistical comparisons for treatment effects *across *subgroups, using a test for heterogeneity or interaction [7,17,18,25-27].

The tension between needing to understand HTE and lacking the statistical power to properly examine it presents difficulties for researchers, clinicians, and patients. While some experts have offered general encouragement to perform more HTE analyses [28,29], the literature is relatively silent on how to manage the risks of over- and under-testing. Kraemer et al. [25,30,31] have suggested a sequential approach that could shed light on possible HTE. Defining treatment *modifiers *as factors that influence the treatment effect size across subgroups, they propose that all RCTs use *exploratory interaction analyses *as a method to generate hypotheses regarding moderators of treatment effects. The presence of strong moderator effects would encourage future researchers to perform adequately powered confirmatory studies stratified prospectively on these moderators. While the proposal of Kraemer et al. makes sense, several small reviews, most published well before the revised CONSORT statement, suggest that testing for HTE is reported in only 25% to 50% of RCTs [18,27,32-35].

We undertook the current review of the prevalence of HTE analyses in a comparatively larger sample of articles published in the general medical literature in order to assess trends over time, examine the appropriateness of the statistical methods used, and explore the predictors of such analyses. A persistently low rate of appropriate HTE or interaction analysis would suggest missed opportunities for identifying HTE.

## Methods

### Overview

We conducted a literature sample of RCTs published in five prominent general medical journals during 1994, 1999, and 2004. The search strategy and abstraction forms incorporated input from a Project Advisory Committee. Human Subjects committee approval was not required. The study was funded by Pfizer, Inc., under a contract to the academic institutions involved. However, the investigators were solely responsible for all aspects of study design, data collection and analysis, and result reporting.

### Data Sources and Searches

**Figure 1:**
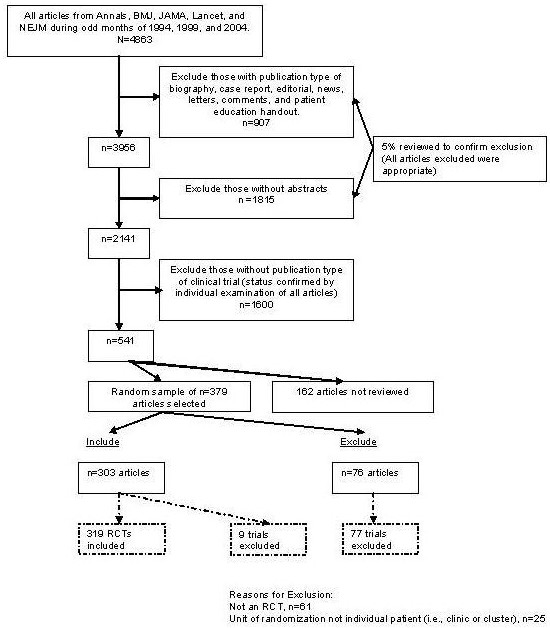
**Article selection for systemic review of heterogeneity of treatment effect in RCTs published in five general medical journals**.

Using PubMed, we searched for RCTs published in the *Annals of Internal Medicine*, *British Medical Journal*, *Journal of the American Medical Association*, *Lancet*, and *New England Journal of Medicine *during odd numbered months in 1994, 1999, and 2004 (Figure 1). These prominent medical journals were selected because they have a broad readership, are the publication venue for many landmark studies, and have disproportionate influence on medical research and practice [36,37]. During the period of interest, 4,863 articles appeared in these journals. Of these, 907 were excluded for having publication types of "biography", "case report", "editorial", "news", "letters", "comments", and "patient education handout". The remaining 3,956 articles were then restricted to include only journal articles and reviews, thereby eliminating all articles without a research abstract (n = 1,815 articles excluded). To assess the validity of these exclusions, one of the investigators reviewed a 5% random sample of the 2,722 excluded articles, resulting in no questionable exclusions. Finally, the remaining 2,141 articles were subdivided into two groups: those with "clinical trial" as a publication type (n = 541) and those without (n = 1,600). The 1,600 articles without clinical trial as publication type were excluded after they were examined by one investigator, and a 10% random sample was additionally examined by a second investigator, without finding any clinical trials. The 541 articles reporting on clinical trials were randomly sorted into 10 batches of 54 articles each (the tenth batch contained 55 articles). Seven batches, or 379 articles, were randomly selected and individually reviewed by two investigators to determine final inclusion status. Discrepancies were resolved in consultation with a third investigator.

### Study Selection

To be included in our sample, a trial had to meet the following criteria: (1) human study population; (2) parallel group RCT (including matched pair trials) or a crossover RCT (including n-of-1 trials); and (3) individual patient or time (treatment episode) within patient (for crossover trials) was the unit of randomization. We excluded trials that used cluster randomization because these trials generally focus on group- or organizational-level treatment effects.

### Data Extraction, Measures, and Hypotheses

All data were abstracted independently by two trained abstractors. Any disagreements were adjudicated by a senior investigator. We used a standard protocol, form, and database that collected the following information: first author's name, article identification number, trial number (if more than one trial was reported in a particular article), condition under study (e.g. cardiovascular disease, cancer), country of first author's institution, continents from which the participants were derived, total number of participants randomized, number and percent of male participants, age of participants (mean, median, standard deviation, range, reported categories), race of participants (number and percent), and number of treatment arms. For each arm, the following information was collected: number of participants; description of treatment provided (i.e. drug, medical device, surgical procedure); and gender, age, and race of participants.

The use (or non-use) of HTE analysis was the primary outcome. In addition, we also examined the presence of subgroup-only analysis, and either subgroup or HTE analysis, as secondary outcomes. Subgroup-only analyses represent missed opportunities on the path to understanding HTE; with minor effort, studies that reported subgroup-only analyses could have conducted formal HTE analyses and provided direct information on HTE. The additional step (of conducting a formal HTE analysis as opposed to a subgroup-only analysis) is important because it will provide important hypothesis-generating information for future studies. We therefore identified all trials as reporting either (1) HTE analysis, utilizing a formal test for heterogeneity or interaction; (2) subgroup-only analysis, with no formal statistical tests for heterogeneity or interaction, or (3) neither. For trials that reported HTE analysis, the covariates examined were also recorded. These covariates were later categorized by one investigator with consultation from a second as needed, into the following categories: age, gender, race/ethnicity, center/trial site/country, individual clinical risk factor, multivariable risk index, co-occurring treatment, comorbidity, and socioeconomic status (income, marital status, and education). Individual clinical risk factors were further categorized as being related to prognosis, treatment responsiveness, or treatment vulnerability [2]. We also coded whether the authors presented information using a Forest Plot (a graph depicting subgroup results as point estimates [boxes] and confidence intervals [lines]) [38].

Potential predictors of HTE and subgroup-only analysis included journal name, year, condition studied, geographic region of the first author's home institution, trial design (either parallel or crossover RCT), and sample size (in quintiles). We expected that different journals might have different reporting policies, possibly influenced by prevailing norms of the country of publication, which in turn might be confounded with the first author's geographic region. We also hypothesized that HTE analysis might increase over time as CONSORT standards were disseminated, and that HTE analysis would increase with sample size. The revised CONSORT standards [18] made a clear distinction between subgroup and HTE analysis, citing a test of interaction as the correct, and stronger, analytic technique. The Statement elaborated on the original 1996 guidelines, and emphasized the incorrectness of comparing subgroup-specific p-values as a method of inferring treatment heterogeneity. Thus, we expected to see in increase in HTE analysis subsequent to 2001, with a concomitant decrease in subgroup analyses. Finally, we hypothesized that trials of common conditions with well-defined prognostic indicators might be more likely to evaluate these indicators.

### Data Analysis

The relationships of HTE reporting with study characteristics were assessed in two-way contingency table analyses by using Pearson Χ^2 ^tests or Fisher's exact test when sample size was small, while trends (where appropriate) were assessed using the Mantel-Hantzel Χ^2 ^test for trend. Logistic regression was used to determine predictors of HTE analysis. Significance of study characteristics in relation to use of HTE analysis was assessed with a Wald Χ^2 ^test. To further explore HTE reporting characteristics, we separately examined the association between study characteristics (other than sample size) and HTE reporting in articles above and below the median sample size (262 subjects). Wald Χ^2 ^tests were used to determine significant differences among these categories. SAS version 9 was used for all computations [39].

We conducted two sensitivity analyses. First, to assess whether our results were sensitive to possible clustering effects arising from multiple trials per article, we re-ran the logistic regression analysis after randomly selecting only one trial from each article that reported on more than one trial. Second, we recalculated reporting of HTE and subgroup analysis after restricting our analysis to those trials meeting specific sample size criteria (overall sample size greater than 250 and at least 100 participants per arm, making reasonably-powered subgroup analyses feasible). Furthermore, we recalculated results for gender and race/ethnicity after restricting to trials with an overall sample size greater than 250, at least 100 participants per arm, and at least 25% participants in the second largest gender or racial/ethnic subgroup.

## Results

Out of the 379 articles identified by our search, 303 met our inclusion criteria and 76 did not (Figure 1). Some articles reported more than one trial, and abstraction occurred on the trial level. Thus, the 303 eligible articles represented 319 eligible RCTs. Twelve articles reported on more than one trial: ten articles reported on two trials each, and two articles reported on four trials each. Eighty-six trials were excluded, including 77 from the 76 ineligible articles, and 9 from the 303 eligible articles. (An eligible article could contain both eligible and ineligible trials; we kept the eligible trials and excluded the ineligible trials in these articles.) The most common reason for exclusion was a trial design other than RCT (n = 61 trials), typically a cohort or case control design. The next most common reason was unit of randomization other than the individual or treatment episode within an individual (n = 25 trials).

Among the 319 eligible trials, cardiovascular (23%) and infectious (22%) diseases were the most frequently studied conditions (Table 1). Sixty-two percent of first authors were from regions outside North America. Parallel group designs were far more prevalent than crossover designs. Trial size ranged from 6 to 41,000 participants (median 262, inter-quartile range 101 to 708). Ninety-two trials (29%) reported HTE analyses that used a test for heterogeneity or interaction; 88 (28%) reported subgroup-only analysis without a statistical test for HTE/interaction; and 139 (44%) reported neither type of analysis. Graphical display of HTE and subgroup effects using Forest Plots was seen in 13/92 (14%) of trials reporting HTE analysis and 5/88 (5.6%) of trials reporting subgroup-only analysis.

**Table 1:** Characteristics of included articles (n = 303) and RCTs described in these articles (n = 319)*

**Characteristic**	**Articles Represented**	**# RCTs Included**
Journal of publication		
Annals	30 (10)	30 (9)

BMJ	43 (14)	47 (15)

JAMA	45 (15)	50 (16)

Lancet	97 (32)	101 (32)

NEJM	88 (29)	91 (29)

Year of publication		
1994	86 (28)	91 (29)

1999	102 (34)	106 (33)

2004	115 (38)	122 (38)

Medical condition under study		
Cardiovascular	69 (23)	74 (23)

Infectious Disease	62 (20)	70 (22)

Cancer	41 (14)	42 (13)

Psychiatry/Neurology	25 (8)	25 (8)

Other	106 (35)	108 (34)

First author's study region		
North America	115 (38)	121 (38)

Other	188 (62)	198 (62)

Study design		
Parallel	-	304 (95)

Crossover	-	15 (5)

Analysis reported		
HTE analysis	-	92 (29)

Subgroup without statistical comparison	-	88 (28)

None	-	139 (44)

Sample size		262 (101 – 708)

	-	[6–41,000]

Abbreviations: RCTs, randomized controlled trials; BMJ, British Medical Journal; JAMA, Journal of the American Medical Association; NEJM, New England Journal of Medicine; HTE, heterogeneity of treatment effects.

*Discrete data are expressed as No. (%); continuous values are presented as median (inter-quartile range) [range].

Among studies reporting HTE analysis on at least one named covariate (n = 91), 47% analyzed one covariate, 26% analyzed 2–4 covariates, 19% analyzed 5–10 covariates, and 8% analyzed more than 10 covariates. Individual risk factors for disease occurrence or progression (e.g. smoking status, creatinine level, CD4 count) were analyzed in 56% of studies, age in 29%, study site or region in 29%, concurrent treatment in 25%, and comorbid medical conditions in 21%. The 51 articles that reported HTE based on individual risk factors for disease occurrence or progression examined a total of 159 variables. Of these variables, 91% were prognostic risk factors, 25% were related to treatment responsiveness, and 4% were factors related to vulnerability to adverse outcomes (some RCTs examined multiple individual clinical risk factors). Treatment by gender interactions were evaluated in 30% of studies in which both genders participated; treatment by race/ethnicity interactions were assessed in 7% of studies involving more than one race/ethnicity. Despite increased recognition of the value of multivariable risk indices in HTE analyses [6,9,40-43] only three studies [44-46] evaluated outcomes of treatment stratified by multivariable risk. When examined by sample size quintile, we found that even studies in the smallest quintile (median = 37 participants) examined up to 9 covariates for HTE.

In the two-way contingency table analysis, performing an HTE analysis was significantly associated with journal of publication (p < 0.05); first author's region (p < 0.01); and sample size (p < 0.0001 for trend) (Table 2). North American journals and first authors were more likely to publish trials with HTE analysis. While the association between HTE analysis and sample size is intuitive, HTE analysis was reported in only 52% of studies within the largest sample size quintile (Table 2). There was also a significant trend toward more HTE analysis over time (p = 0.047). Subgroup analysis only (without an appropriate statistical test for heterogeneity or interaction) was observed in 29% of parallel group trials and in no crossover trials. In the two-way contingency table analysis, subgroup analysis only was not significantly associated with any of the study characteristics (data not shown).

**Table 2:** HTE reporting by study characteristics (n = 319 RCTs in 303 articles)

**Characteristic**	**N**	**No. (%) Reporting HTE**	**No. (%) Reporting Either subgroup or HTE**
Journal of publication		†	†
Annals	30	11 (37)	16 (53)

BMJ	47	9 (19)	18 (38)

JAMA	50	21 (42)	33 (66)

Lancet	101	21 (21)	53 (53)

NEJM	91	30 (33)	60 (66)

Year of publication			
1994	91	20 (22)	47 (52)

1999	106	30 (28)	62 (58)

2004	122	42 (34)	71 (58)

Medical condition under study			‡
Cardiovascular	74	21 (28)	43 (58)

Infectious disease	70	25 (36)	48 (69)

Cancer	42	15 (36)	30 (71)

Psychiatry/Neurology	25	7 (28)	12 (48)

Other	108	24 (22)	47 (44)

First author's study region		‡	‡
North America	121	47 (39)	80 (66)

Other	198	45 (23)	100 (51)

Study design			∥
Parallel	304	89 (29)	177 (58)

Crossover	15	3 (20)	3 (20)

Sample size		**	**
Quintile 1 (median = 37)	64	9 (14)	18 (28)

Quintile 2 (median = 124)	64	7 (11)	29 (45)

Quintile 3 (median = 263)	64	22 (34)	45 (70)

Quintile 4 (median = 549)	64	21 (33)	40 (63)

Quintile 5 (median = 1560)	63	33 (52)	48 (76)

Abbreviations: HTE, heterogeneity of treatment effects; RCTs, randomized controlled trials; BMJ, British Medical Journal; JAMA, Journal of the American Medical Association; NEJM, New England Journal of Medicine.

^†^p < 0.05 by Pearson Chi-square test

^‡^p < 0.01 by Pearson Chi-square test

^§^p < 0.05 by Fisher's exact test

^∥^p < 0.01 by Fisher's exact test

^¶^p < 0.05 by Mantel-Hantzel test for trend

**p < 0.0001 by Mantel-Hantzel test for trend

Using multiple logistic regression to examine trial year, journal of publication, clinical condition, first author's trial region, and sample size, only sample size and journal of publication were significant predictors of HTE analysis; we found no significant trend over time (p = 0.52) (Table 3). The adjusted odds ratios comparing the top quintile of sample size (median = 1560) to the bottom quintile (median = 37) was 7.5 (95% confidence interval, 2.9–19.3). For journal, the adjusted odds ratio for *Annals of Internal Medicine *was 4.2 (95% CI, 1.2–15.1) and for *JAMA *was 4.4 (95% CI, 1.4–13.5), with *BMJ *as the reference group. Restricting the analysis to trials above the median sample size (n ≥ 262 subjects), 42% of trials performed HTE analysis (35% in the 3^rd ^quartile, 48% in the 4^th ^quartile; data not shown in tabular form). Random selection of only one trial per article (to avert any possible clustering effect) did not materially alter the results of the logistic regression analysis.

**Table 3:** Logistic regression results examining predictors of HTE or subgroup analysis

	**Predict HTE analysis**
**Condition**	OR (95% CI)*
Journal of publication	†
BMJ	1.00 [reference]

Annals	4.22 (1.18–15.08)

JAMA	4.40 (1.43–13.53)

Lancet	1.44 (0.55–3.77)

NEJM	2.36 (0.85–6.56)

Year of publication	
2004	1.00 [reference]

1999	0.88 (0.47–1.66)

1994	0.83 (0.41–1.67)

Medical condition under study	
Other	1.00 [reference]

Cardiovascular	1.07 (0.50–2.32)

Infectious disease	1.38 (0.66–2.89)

Cancer	1.13 (0.47–2.71)

Psychiatry/Neurology	2.85 (0.91–8.92)

First author's study region	
Other	1.00 [reference]

North America	1.23 (0.67–2.29)

Sample size	‡
Quintile 1 (median = 37)	1.00 [reference]

Quintile 2 (median = 124)	0.72 (0.24–2.14)

Quintile 3 (median = 263)	4.17 (1.62–10.74)

Quintile 4 (median = 549)	3.08 (1.20–7.91)

Quintile 5 (median = 1560)	7.55 (2.95–19.33)

Abbreviations: HTE, heterogeneity of treatment effects; OR, odds ratio; CI, confidence interval; BMJ, British Medical Journal; JAMA, Journal of the American Medical Association; NEJM, New England Journal of Medicine

*Adjusted for all other variables in the model

^†^Significant at p < 0.05 for a combined Wald Chi-square test

^‡^Significant at p < 0.001 for a combined Wald Chi-square test

In the sensitivity analysis, there were 153 studies with sample size of at least 250 with a minimum of 100 subjects per trial arm. Sixty-one of these trials (40%) reported HTE analysis, 47 (31%) reported subgroup analysis only, and 45 studies (29%) did not report either type of analysis. Among 104 trials with at least 25 male subjects and 25 female subjects per arm, 14 trials (13.5%) examined gender for HTE, compared with 3/33 (9%) among those not meeting these minimal sample size criteria. Only 5/34 (15%) of trials meeting minimal sample size criteria for race/ethnicity (see Methods) conducted an HTE analysis with respect to race/ethnicity.

## Discussion

This review of 319 RCTs published in five prominent general medical journals is the most comprehensive to date, and the only one that examines trends of HTE reporting over time. The results suggest that reporting on HTE occurs in less than one-third of studies published in prominent general medical journals, and were only marginally better in 2004 than in 1994. Overall, less than one-third of studies in our sample reported HTE analysis, a result consistent with previous, less comprehensive reviews. Another 28% reported subgroup-only analyses without formal statistical tests for HTE/interaction.

These trials are missed opportunities. With minimal additional effort, they could have added statistical tests for HTE or interaction in addition to the subgroup results that they reported, nearly doubling the proportion of HTE analyses and enriching the literature with much-needed HTE information. Such tests are critical for appropriate interpretation of results, as differences in subgroup-specific point estimates are meaningful only when evaluated alongside their corresponding confidence intervals.

Considering both HTE analyses and subgroup-only analyses, 57% of the trials in our review reported some kind of subgroup analysis, a proportion that increased to about three-quarters if we examine only the largest trials. Previous research reported equal [34] or higher [27,32,33,35] proportions, possibly because of restriction to trials of a minimum sample size, a specific discipline, or a specific journal.

**Figure 2:**
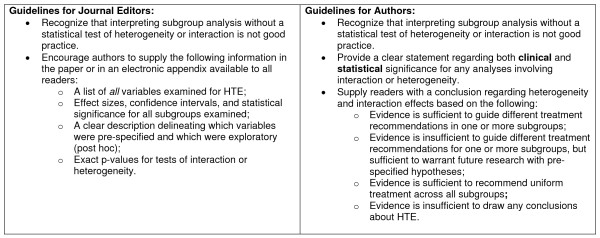
**Guidelines for Journal Editors and Authors for Reporting and Interpreting HTE Analyses**.

The biostatistical literature tends to view subgroup analysis skeptically, often citing the dual problems of multiple statistical comparisons and low power [16,47]. This view is likely reinforced by the impression that the analyses themselves are poorly performed. Our results support this judgment. Wang et al. [35] recently outlined detailed guidelines for reporting subgroup analyses in manuscripts, including specific information that authors should include, by section. We present complementary guidelines in Figure 2, and emphasize the role of journal editors in setting appropriate standards for subgroup analysis reporting. There are three major principles to our guidelines. The first and second underscore the CONSORT recommendations [18] that (1) proper HTE analysis requires statistical tests of interaction or heterogeneity and (2) that all variables examined for HTE should be labeled as prespecified or post hoc and reported in the body of the paper or in an electronic appendix. Finally, we advocate for authors explicitly considering the clinical and statistical significance of results obtained, and provide recommendations for future research and clinical practice. In addition, when the purpose of HTE analysis is hypothesis generation, it may make sense to accept a more lenient standard of statistical significance (i.e. 0.10). Reporting only subgroup-specific effects without an appropriate test of heterogeneity or interaction can be misleading [24]. Yet identifying potential moderators of treatment effects is critical to patient-centered, individualized care [2,8,25,48]. Inconsistent reporting of subgroup analysis not only impairs recognition of patients who may respond better or worse than average, but also impedes hypothesis generation and stifles future research. Although tests for heterogeneity and interaction have low power for detecting modest but potentially clinically important subgroup differences, they represent a conservative approach and provide a brake on the tendency to over-interpret observed subgroup differences. However, it is important to conduct HTE analysis with caution and not over interpret the results. It is necessary to recognize that post hoc HTE analyses are for hypothesis generation and to aid in the design of future confirmatory studies, that significant effects may be a result of intra-individual variability, and that the results of such analyses should not be used to promote different treatment recommendations.

Among trials that explored heterogeneity of treatment effects, clinical prognostic factors were evaluated frequently; age, gender, and site factors less often; and race/ethnicity rarely. Even among those trials with a sample size adequate for exploring HTE by race/ethnicity, only 7% of trials did so. The limited attention to race/ethnicity is puzzling for two reasons. First, the literature provides examples in which race/ethnicity influences baseline risk of a disease [49], responsiveness to treatment [50], and vulnerability to adverse outcomes [51]. Second, growing interest in genomics might be expected to stimulate interest in the treatment-modifying effects of genetic proxies, including imperfect ones like race/ethnicity. Consistent with Parker et al. [27], we found little use of multivariate risk stratification, an approach that may greatly increase statistical power for detecting HTE [48].

More frequent HTE analysis in North American journals may reflect differences in biostatistical perspectives or biomedical culture. The dominant norm tends to be more conservative in Europe and especially in Britain, perhaps as a result of public payment for care, which demands a higher standard of evidence before treatments are widely accepted and delivered [52-54]. The trend for increased HTE analysis between 1994 and 2004 may be attributable to the revised CONSORT recommendations and a growing awareness of the potential of such analyses. The relatively infrequent use of Forest Plots, even among studies reporting HTE analysis, is regrettable because these plots are a simple, compact, and readily understood method of presenting potential moderators.

Our results should be interpreted in light of several limitations. First, we reviewed a limited number of trials. It is possible that subgroup-specific trials were published in other journals, or during months or years that we did not sample. However, our sample included five large-circulation, high-impact journals with strict peer-review standards, so our sample should be representative of well-designed clinical trials that generalist clinicians are most likely to read and that major news media are most likely to publicize. Second, it is possible that some trials conducted HTE analysis but did not report it because the results were not statistically significant. However, even non-significant data are useful for the purposes of hypothesis generation and, arguably, authors should report any HTE analysis, significant or not, and especially when the analysis is pre-specified. Third, our standards for HTE reporting may not reflect journals' own standards for HTE reporting. A more conservative statistical review process may result in reduced HTE reporting, regardless of the analysis actually conducted in the study. Finally, our recommendations for authors and editors are based on an informal procedure, and should be interpreted in light of this limitation. Further refinement of the recommendations may be necessary before adoption by editors and authors.

## Conclusion

Our findings indicate that HTE reporting in the general medical literature is neither rigorous nor routine. Given the increasing recognition of HTE [2,3,25], it may be time to develop standards for reporting of exploratory and confirmatory HTE analysis. In 1994, the National Institutes of Health mandated the inclusion of women and racial/ethnic minorities in research populations and, in 2000, supplemented that recommendation with guidelines regarding the reporting of subgroup-specific results of Phase III Clinical Trials [55]. Although these guidelines included a recommendation that investigators report both significant and non-significant results, our data show only modest progress toward that goal. Highlighting variables that deserve further exploration is a first step in identifying groups that may or may not respond better to a given therapy [56]. Because trials in more responsive subgroups have lower sample size requirements, identifying these groups through exploratory subgroup analysis could facilitate relatively cost-effective confirmatory trials. An iterative process of exploratory followed by confirmatory HTE analysis may not only quicken the cycle of discovery but also inform clinical judgment.

## Abbreviations

HTE: heterogeneity of treatment effects; RCT: randomized controlled trials; ITE: individual treatment effect; CONSORT: Consolidated Standards of Reporting Trials; BMJ: British Medical Journal; JAMA: Journal of the American Medical Association; NEJM: New England Journal of Medicine.

## Competing interests

The authors declare that they have no competing interests.

This work was supported by a grant from Pfizer, Inc.

Dr. Elmore is supported by a Public Health Research grant from the National Institutes of Health (#5 K05 CA104699-04). Dr. Kravitz holds a K24 Midcareer Research and Development Award (#5 K24 MH072756-03) from the National Institute of Mental Health.

## Authors' contributions

The authors co-developed the ideas in the manuscript. NG drafted the manuscript and RK, ND, JE, and TG reviewed and revised the manuscript. DL provided statistical analysis support. NG had full access to all of the data in the study and takes responsibility for the integrity of the data and the accuracy of the data analysis. All authors read and approved the final manuscript.
